# Retinal vasculitis after intravitreal aflibercept 8 mg for neovascular age-related macular degeneration

**DOI:** 10.1007/s10384-024-01107-w

**Published:** 2024-08-20

**Authors:** Hidetaka Matsumoto, Junki Hoshino, Saki Numaga, Kaori Mimura, Yosuke Asatori, Hideo Akiyama

**Affiliations:** Department of Ophthalmology, Gunma University Graduate School of Medicine, 3-39-15 Showa-machi, Maebashi, 371-8511 Gunma Japan

**Keywords:** Age-related macular degeneration, Anti-vascular endothelial growth factor, Aflibercept 8 mg, Retinal vasculitis, Intraocular inflammation

## Abstract

**Purpose:**

To evaluate short-term outcomes of intravitreal injection of aflibercept 8 mg for neovascular age-related macular degeneration (nAMD).

**Study design:**

Retrospective, interventional case series.

**Methods:**

We retrospectively studied 35 eyes of 34 consecutive patients with nAMD, assessing best-corrected visual acuity (BCVA), foveal thickness (FT), and central choroidal thickness (CCT) before and 4 weeks after the initial intravitreal dose of aflibercept 8 mg. The rate of achieving a dry macula and the incidence of intraocular inflammation (IOI) at week 4 were also determined.

**Results:**

BCVA showed significant improvement, with significant reductions in FT and CCT 4 weeks after the initial injection of aflibercept 8 mg (all *P* < 0.01), with a dry macula being achieved in 20 eyes (57.1%). However, 3 eyes (8.6%) developed non-infectious IOI associated with retinal vasculitis, an adverse event not reported previously. The IOI in these eyes was relatively mild and treated with a posterior subtenon injection of triamcinolone acetonide with or without betamethasone eye drops, resulting in amelioration of IOI without any visual loss.

**Conclusions:**

Intravitreal aflibercept 8 mg appears to be effective for improving visual acuity and ameliorating exudative changes in eyes with nAMD. However, special attention should be given to the potential development of IOI associated with retinal vasculitis.

**Supplementary Information:**

The online version contains supplementary material available at 10.1007/s10384-024-01107-w.

## Introduction

Intravitreal injection of an anti-vascular endothelial growth factor (VEGF) drug is currently the first line treatment for neovascular age-related macular degeneration (nAMD) [1]. Several anti-VEGF drugs such as ranibizumab, aflibercept 2 mg, brolucizumab, and faricimab have been developed and used clinically. Aflibercept 2 mg is widely employed globally and served as a control drug in the phase 3 clinical trials, the HAWK and HARRIER trials for brolucizumab, and the TENAYA and LUCERNE trials for faricimab [2–5].

In the HAWK and HARRIER trials, it was demonstrated that, in terms of improvement in best-corrected visual acuity (BCVA) scores, injections of brolucizumab every 12 weeks were non-inferior to injections of aflibercept 2 mg every 8 weeks [2, 3]. Moreover, due to the marked fluid control effect exerted by brolucizumab, the brolucizumab group showed significantly greater reductions in central retinal thickness than the aflibercept 2 mg group [2, 3]. However, the brolucizumab group had higher incidences of intraocular inflammation (IOI), including retinal vasculitis and retinal vascular occlusion, than the aflibercept 2 mg group, raising concerns about brolucizumab use [6, 7].

On the other hand, in the TENAYA and LUCERNE trials, injections of faricimab every 8, 12 or 16 weeks demonstrated to be non-inferior to injections of aflibercept 2 mg every 8 weeks in terms of improvement in BCVA scores and reducing central subfield thickness [4, 5]. In the evaluation up to week 12 of the loading phase, the fluid control effect of faricimab was significantly higher than aflibercept 2 mg [8, 9]. Additionally, there were no significant differences in safety profiles between faricimab and aflibercept 2 mg [4, 5]. Although IOI was observed in a few cases in both groups, neither treatment-related retinal vasculitis nor retinal vascular occlusion was observed [4, 5].

In 2023, aflibercept 8 mg was launched as a new anti-VEGF drug following a phase 3 clinical trial, the PULSAR trial, with aflibercept 2 mg serving as the control drug [10]. The first-year results of the PULSAR trial demonstrated injections of aflibercept 8 mg every 12 or 16 weeks to be non-inferior to injections of aflibercept 2 mg every 8 weeks in terms of improvements in BCVA and central retinal thickness [10]. Additionally, in the evaluation up to week 16 of the loading phase, the fluid control effect of aflibercept 8 mg was significantly superior to aflibercept 2 mg [10]. Among the safety profiles, there were no significant differences between aflibercept 2 mg and 8 mg, with the incidence of iritis and vitritis being less than 1% in both groups, and no cases developed retinal vasculitis [10]. However, when we used aflibercept 8 mg for nAMD in clinical practice, we encountered multiple cases of IOI associated with retinal vasculitis, an adverse event not reported previously. In this study, we retrospectively evaluated the short-term outcomes of intravitreal aflibercept 8 mg for nAMD in real-world settings.

## Materials and methods

We obtained approval for this study, which complied with the guidelines of the Declaration of Helsinki, from the Institutional Review Board of Gunma University Hospital. We used an opt-out informed consent protocol due to the retrospective design of the study. We studied 35 eyes of 34 patients with nAMD. During the period from April 24th, 2024, through May 31st, 2024, the patients received an initial treatment with intravitreal aflibercept 8 mg at Gunma University Hospital.

Before starting any treatment for nAMD, all patients underwent complete ophthalmological examinations, including slit-lamp biomicroscopy with a noncontact fundus lens (SuperField lens; Volk Optical Inc), color fundus photography (Canon CX-1; Canon), ultra-widefield color fundus imaging (Optos 200Tx, Optos), fluorescein angiography (FA) and indocyanine green angiography (ICGA) (Spectralis HRA + OCT; Heidelberg Engineering, California; Optos), as well as swept-source optical coherence tomography (OCT) (DRI OCT-1 Triton; Topcon Corp, and PLEX Elite 9000; Carl Zeiss Meditec). For the OCT examination, we obtained B-mode images of the horizontal and vertical line scans (12 mm) through the fovea as well as 12 radial scans (9 mm) centered on the fovea employing the DRI OCT-1 Triton. Then, we performed OCT angiography (OCTA) volume scanning, i.e., 300 × 300 pixels in the 3 × 3 mm area demonstrated by the PLEX Elite 9000. The OCTA thus performed was based on an optical microangiography algorithm. The diagnostic criteria for nAMD were based on a previous report detailing nAMD nomenclature [11]. The presence of polypoidal lesions was evaluated on ICGA and B-mode OCT images, i.e., polyp-like choroidal vessel dilation on ICGA and sharply peaked retinal pigment epithelium (RPE) detachment on B-mode OCT.

All eyes were treated with an initial intravitreal injection of aflibercept 8 mg (8 mg/0.07mL). After 4 weeks, if non-infectious IOI developed, aflibercept 8 mg therapy was discontinued and posterior subtenon injection of triamcinolone acetonide (30 mg/0.75mL) with or without 0.1% betamethasone eye drops was administered according to the treatment protocol for brolucizumab-related IOI [12, 13]. Otherwise, a second intravitreal aflibercept 8 mg injection was given. Additionally, if patient consent was obtained, FA and ICGA were performed again for those who developed IOI.

BCVA, foveal thickness (FT), and central choroidal thickness (CCT) were examined at each visit. BCVA was determined with manifest refraction and recorded as decimal values, then converted to the logarithm of the minimal angle of resolution (logMAR) units. FT and CCT were measured on B-scan OCT images employing the computer-based caliper measurement tool in the OCT system. FT was, by definition, the distance between the internal limiting membrane and the RPE surface at the fovea. FT included any intraretinal and subretinal fluid. CCT was defined as the distance between Bruch’s membrane and the margin of the choroid and sclera under the fovea. Dry macula was defined as the macula showing no evidence of intraretinal, subretinal, or sub-RPE fluid accompanied by either no or diminishing hemorrhage.

For statistical analyses, the Wilcoxon signed-rank test was applied for comparing the differences between BCVA, FT and CCT at baseline versus 4 weeks after initial intravitreal aflibercept 8 mg. The data analyses were performed employing Excel (Microsoft) with add-in software Statcel4 [14]. A value of *P* < 0.05 was considered to indicate a statistically significant difference. All data are presented as the average ± standard deviation.

## Results

The subjects were 35 eyes of 34 patients (23 eyes of 23 men; 12 eyes of 11 women, average age: 76.5 ± 9.4 years) with nAMD. Macular neovascularization (MNV) subtypes were: type 1: 10 eyes (28.6%), polypoidal choroidal vasculopathy (PCV): 16 eyes (45.7%), type 2: 4 eyes (11.4%), mixed type 1 and type 2: 3 eyes (8.6%), type 3: 2 eyes (5.7%). Among the 35 eyes studied, 18 (51.4%) were treatment-naïve, while 17 (48.6%) had switched from other anti-VEGF drugs, including aflibercept 2 mg in 7, brolucizumab in 2, and faricimab in 8 eyes. Prior to the switch, 5 eyes had a history of brolucizumab-related IOI, and 1 eye had developed faricimab-related IOI. The baseline demographic and clinical characteristics of our nAMD patients treated with intravitreal aflibercept 8 mg are presented in Table 1.

BCVA was 0.27 ± 0.34 logMAR units at baseline and showed significant improvement to 0.22 ± 0.32 after 4 weeks (*P* < 0.01). FT was 239 ± 105 μm at baseline and was significantly decreased to 178 ± 71 μm after 4 weeks (*P* < 0.01). Moreover, CCT was 165 ± 89 μm at baseline and showed a significant reduction to 143 ± 79 μm after 4 weeks (*P* < 0.01). Dry macula was confirmed in 20 eyes (57.1%) 4 weeks after the initial injection of aflibercept 8 mg.

**Fig. 1:**
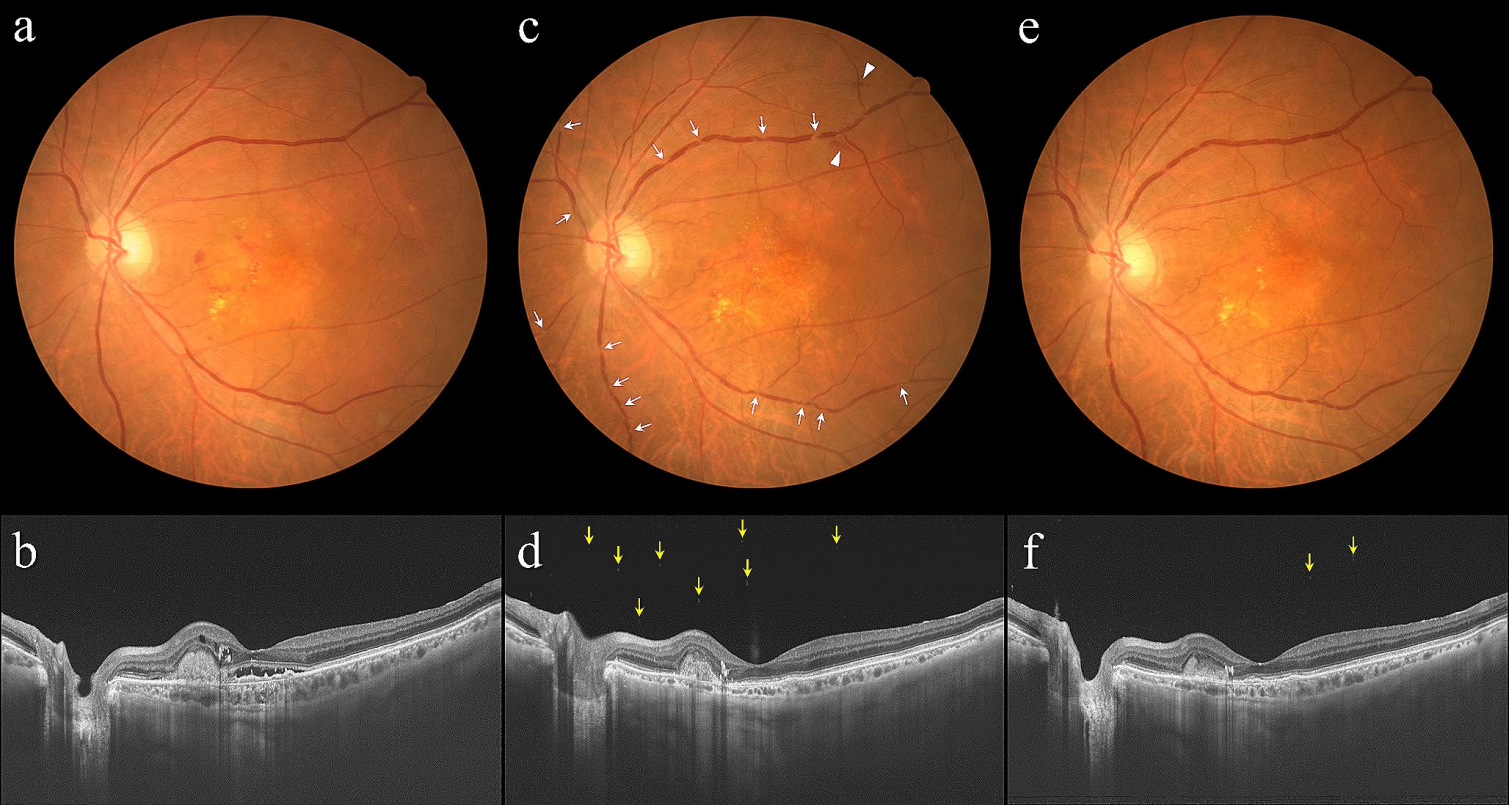
Images of the left eye of an 82-year-old man with treatment-naïve neovascular age-related macular degeneration associated with mixed type 1 and type 2 macular neovascularization. At baseline, best-corrected visual acuity (BCVA) was 0.6 (0.22 logarithm of the minimum angle of resolution (logMAR) units). (**a**) Color fundus photograph shows retinal pigment epithelium (RPE) degeneration accompanied by subretinal hemorrhage and hard exudate at the macular area. The retinal vessels appear normal. (**b**) Optical coherence tomography (OCT) shows a shallow irregular RPE elevation and subretinal hyperreflective material, reflecting mixed type 1 and type 2 macular neovascularization, accompanied by subretinal and intraretinal fluid. The foveal thickness and central choroidal thickness are 193 μm and 214 μm, respectively. Four weeks after the initial injection of aflibercept 8 mg, BCVA of the left eye is 0.6 (0.22 logMAR units). (**c**) Color fundus photograph shows multiple sites of localized narrowing of the retinal vessels, especially retinal veins (arrows), and mild intraretinal hemorrhage (arrow heads). Subretinal hemorrhage and hard exudate at the macular area have decreased. (**d**) OCT shows several vitreous cells (arrows). Shallow irregular RPE elevation and subretinal hyperreflective material have diminished, and subretinal and intraretinal fluid have been resolved. The foveal thickness and central choroidal thickness are 145 μm and 87 μm, respectively. Aflibercept 8 mg therapy was discontinued due to the development of intraocular inflammation associated with retinal vasculitis. Two weeks after a posterior subtenon injection of triamcinolone acetonide (30 mg/0.75mL) with 0.1% betamethasone eye drops, BCVA of the left eye is 0.8 (0.10 logMAR units). (**e**) Color fundus photograph shows a reduction in localized narrowing of the retinal vessels, with disappearance of intraretinal hemorrhage. (**f**) OCT shows a reduced number of vitreous cells (arrows)

**Fig. 2:**
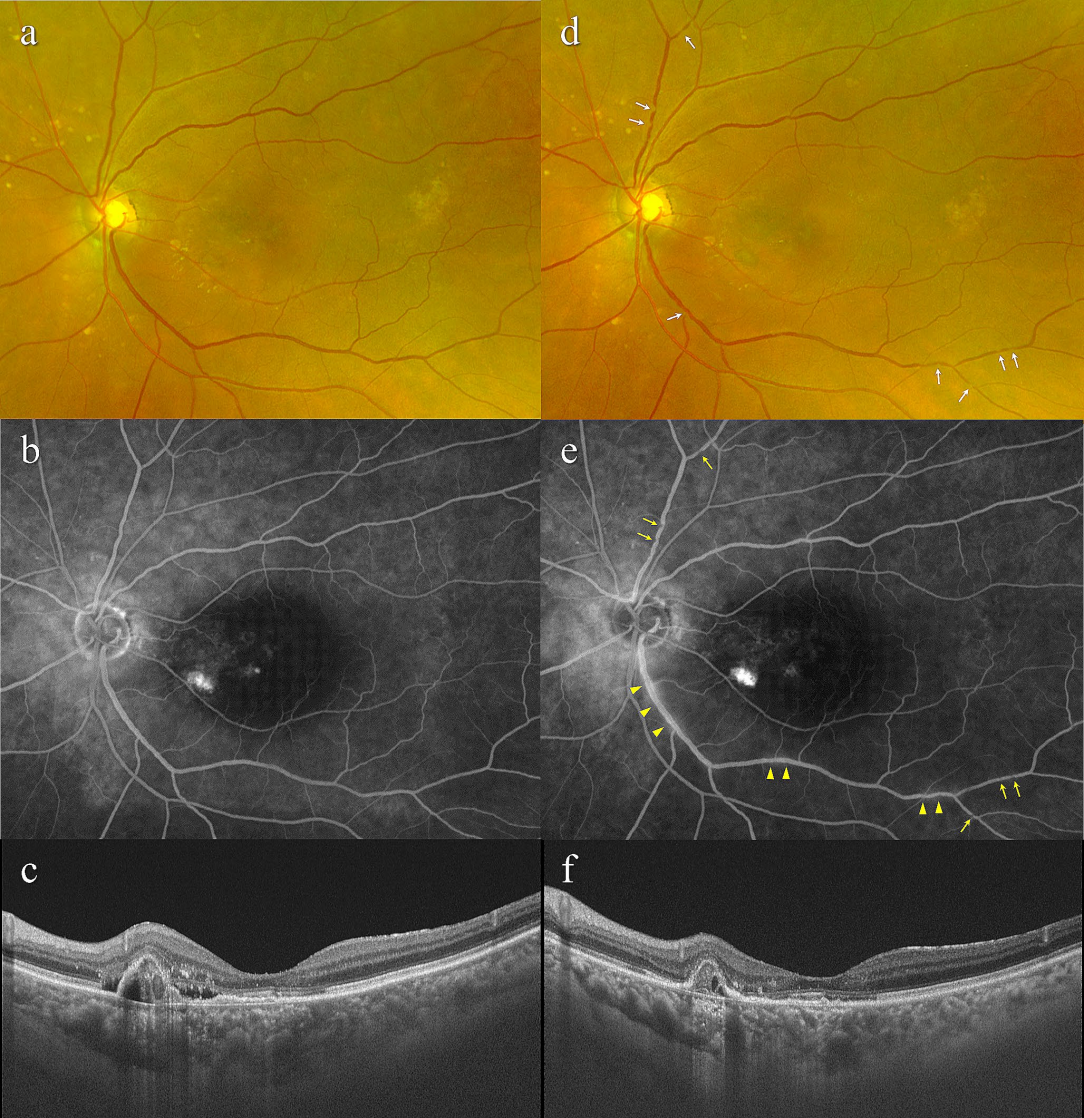
Images of the left eye of a 79-year-old woman with treatment-naïve neovascular age-related macular degeneration associated with polypoidal choroidal vasculopathy. At baseline, best-corrected visual acuity (BCVA) was 0.8 (0.10 logarithm of the minimum angle of resolution (logMAR) units). (**a**) Color fundus photograph shows retinal pigment epithelium (RPE) degeneration at the macular area. The retinal vessels appear normal. (**b**) Fluorescein angiography demonstrates mild leakage and window defects at the macular area. The retinal vessels appear normal. (**c**) Optical coherence tomography (OCT) shows a shallow irregular RPE elevation (double layer sign) and protruding RPE detachment, reflecting a branching neovascular network and polypoidal lesion, accompanied by subretinal and sub-RPE fluid. The foveal thickness and central choroidal thickness are 129 μm and 442 μm, respectively. Four weeks after initial injection of aflibercept 8 mg, BCVA of the left eye is 0.8 (0.10 logMAR units). (**d**) Color fundus photograph shows multiple sites of localized narrowing of the retinal vessels, especially retinal veins (arrows). (**e**) Fluorescein angiography reveals multiple sites of localized narrowing of the retinal vessels, especially retinal veins (arrows), and mild leakage from inferior temporal arcade retinal vein (arrow heads). (**f**) OCT shows that shallow irregular RPE elevation and protruding RPE detachment have diminished, and there is resolution of subretinal and sub-RPE fluid. The foveal thickness and central choroidal thickness are 118 μm and 384 μm, respectively. Aflibercept 8 mg therapy was discontinued due to the development of intraocular inflammation associated with retinal vasculitis. Subsequently, a posterior subtenon injection of triamcinolone acetonide (30 mg/0.75mL) was administered

Three eyes (8.6%) of 3 patients developed non-infectious IOI. The first patient was an 82-year-old man with treatment-naïve mixed type 1 and type 2 MNV, showing retinal vasculitis and vitritis. Four weeks after the initial injection of aflibercept 8 mg, multiple sites of localized narrowing of the retinal vessels, especially retinal veins, and mild intraretinal hemorrhage were observed. Moreover, vitreous cells were detected by OCT (Fig. 1 and Supplemental Fig. 1). The second patient was a 79-year-old woman, with treatment-naïve PCV, who developed retinal vasculitis. Four weeks after the initial aflibercept 8 mg injection, multiple sites of localized narrowing of the retinal vessels, especially retinal veins, were seen. Additionally, FA revealed mild leakage from retinal veins (Fig. 2). The third patient was a 78-year-old man with previously treated PCV, presenting with mild retinal vasculitis. This patient had a history of faricimab-related IOI and had been given 5 monthly injections of aflibercept 2 mg without developing IOI. Four weeks after switching to aflibercept 8 mg, multiple sites of localized narrowing of the retinal vessels, especially retinal veins, were observed (Supplemental Fig. 2). None of these patients reported symptoms attributable to the IOI or experienced visual decline 4 weeks after the initial intravitreal aflibercept 8 mg dose. IOI in these cases was ameliorated with a subtenon injection of triamcinolone acetonide (30 mg/0.75 mL) with or without 0.1% betamethasone eye drops (Fig. 1 and Supplemental Fig. 2). Five eyes that had previously shown brolucizumab-related IOI did not develop any form of IOI within 4 weeks after the initial injection of aflibercept 8 mg.

**Table 1:** Baseline demographic and clinical characteristics of nAMD patients treated with intravitreal injection of aflibercept 8 mg

Number of eyes	35	
Number of patients	34	
Age (years)	76.5 ± 9.4	
Male	23 (67.6%)	
Type of macular neovascularization	Type 1	10 (28.6%)
	PCV	16 (45.7%)
	Type 2	4 (11.4%)
	Mixed type 1 and type 2	3 (8.6%)
	Type 3	2 (5.7%)
Treatment-naïve	18 (51.4%)	
History of brolucizumab-related IOI	5 (14.3%)	
History of faricimab-related IOI	1 (2.9%)	
Best-corrected visual acuity (logMAR)	0.27 ± 0.34	
Foveal thickness (µm)	239 ± 105	
Central choroidal thickness (µm)	165 ± 89	

nAMD: neovascular age-related macular degeneration, PCV: polypoidal choroidal vasculopathy, IOI: intraocular inflammation

## Discussion

We treated 35 consecutive eyes with nAMD using intravitreal aflibercept 8 mg and retrospectively evaluated the short-term outcomes. BCVA showed significant improvement, with significant reductions in FT and CCT 4 weeks after the initial injection of aflibercept 8 mg, achieving a dry macula in 20 eyes (57.1%). However, 3 eyes (8.6%) developed non-infectious IOI associated with retinal vasculitis, an adverse event not reported previously, even in clinical trials for aflibercept 8 mg [10, 15]. IOI in these cases was ameliorated with a posterior subtenon injection of triamcinolone acetonide with or without betamethasone eye drops.

Many reports describe non-infectious IOI following intravitreal injections of anti-VEGF drugs, especially brolucizumab [6, 7, 13, 16–18]. In the HAWK and HARRIER trials, IOI was observed in 4.6% of cases, and when limited to Japanese subjects, the affected proportion was 12.9% [7, 19]. Additionally, retinal vasculitis was observed in 3.3% of cases overall and in 9.9% of the Japanese subjects [7, 19]. The precise mechanism underlying the development of IOI is not fully understood. However, it is thought to possibly be attributable to a type III hypersensitivity reaction involving anti-drug antibodies or inflammatory reactions caused by vascular endothelial cell damage due to the anti-VEGF effect [20–22]. Regarding anti-drug antibodies, some patients may inherently possess antibodies against anti-VEGF drugs, or antibodies may be produced after intravitreal injection of anti-VEGF drugs. This can trigger an immune response against the intravitreally administered anti-VEGF drugs, leading to IOI such as iritis, vitritis, and retinal vasculitis. As for vascular endothelial cell damage, anti-VEGF drugs are used to suppress neovascularization activity by blocking VEGF which is essential for maintaining various physiological functions, including vascular endothelial cell homeostasis [23]. Therefore, marked prolonged blocking of VEGF by anti-VEGF drugs may result in damage to normal vascular endothelial cells. This damage may then lead to inflammatory cells, such as monocytes, migrating to affected endothelial cells [24], thereby narrowing the vascular lumen. This might explain the localized narrowing of retinal vessels observed in our present study. Moreover, pro-inflammatory cytokines released by migrating monocytes and monocyte-derived macrophages have the potential to compromise the barrier function of endothelial cells [24], resulting in leakage from retinal vessels as observed in FA.

In this study, there were 5 eyes and 1 eye, respectively, with a history of IOI following previous injections of brolucizumab or faricimab. Among the cases with prior brolucizumab-related IOI, none exhibited IOI after the initial intravitreal aflibercept 8 mg. However, the case with prior faricimab-related IOI developed IOI associated with retinal vasculitis after the initial injection of aflibercept 8 mg. This case had been given 5 monthly injections of aflibercept 2 mg following the faricimab-related IOI without IOI development. Relative to aflibercept 2 mg at a molar dose of 1 (representing anti-VEGF-A efficacy), those of brolucizumab, faricimab and aflibercept 8 mg were 13.8, 2.4, and 4 times, respectively [25]. These findings suggest that IOI associated with retinal vasculitis following intravitreal aflibercept 8 mg may arise more from endothelial cell damage due to potent VEGF inhibition than from a hypersensitivity reaction associated with drug antibodies. The absence of aflibercept 8 mg-related IOI in the cases with a history of brolucizumab-related IOI, and the development of IOI associated with retinal vasculitis in the case switching from the 2 mg to the 8 mg dose of aflibercept with a history of faricimab-related IOI, are consistent with the varying strengths of each drug’s anti-VEGF-A effect.

This study has several limitations, including the retrospective single-center design and the rather small number of study subjects. Moreover, the evaluation was restricted to the treatment outcomes of initial intravitreal aflibercept 8 mg for nAMD. The HAWK and HARRIER trials, as well as our real-world clinical experience, indicate a higher incidence of brolucizumab-related IOI during the loading phase of treatment [7, 13]. Therefore, careful monitoring is warranted for cases starting treatment with aflibercept 8 mg. Larger and longer-term studies are needed to assess the frequency of IOI associated with aflibercept 8 mg. The IOI associated with retinal vasculitis observed in this study was relatively mild compared to that following brolucizumab administration; however, anti-inflammatory treatment was administered with posterior subtenon injection of triamcinolone acetonide with or without betamethasone eye drops in accordance with the established treatment protocol for brolucizumab-related IOI [12, 13]. Further research is necessary to determine the optimal treatment approach for aflibercept 8 mg-related IOI associated with retinal vasculitis. All subjects were Japanese, hence our results might not be generalizable to nAMD in Caucasians and other racial or ethnic groups.

In conclusion, the initial injection of aflibercept 8 mg was effective for improving visual acuity and ameliorating exudative changes in eyes with nAMD. However, we encountered an adverse event, IOI associated with retinal vasculitis, which has not been reported previously. This complication showed amelioration in response to a subtenon injection of triamcinolone acetonide with or without betamethasone eye drops. Further investigation is necessary to determine the incidence and optimal management strategies for this condition. There is a possibility that vascular endothelial cell damage associated with high-concentration intravitreal anti-VEGF therapy contributes to retinal vasculitis.

## Electronic supplementary material

Below is the link to the electronic supplementary material.

Supplementary Material 1

Supplementary Material 2

Supplementary Material 3
