# Supplementary figures and images for: Retinal vasculitis after intravitreal aflibercept 8 mg for neovascular age-related macular degeneration
Source: Jpn J Ophthalmol. 2024 Aug 20;68(5):531–7. doi: 10.1007/s10384-024-01107-w (PMC11420316; doi:10.1007/s10384-024-01107-w)

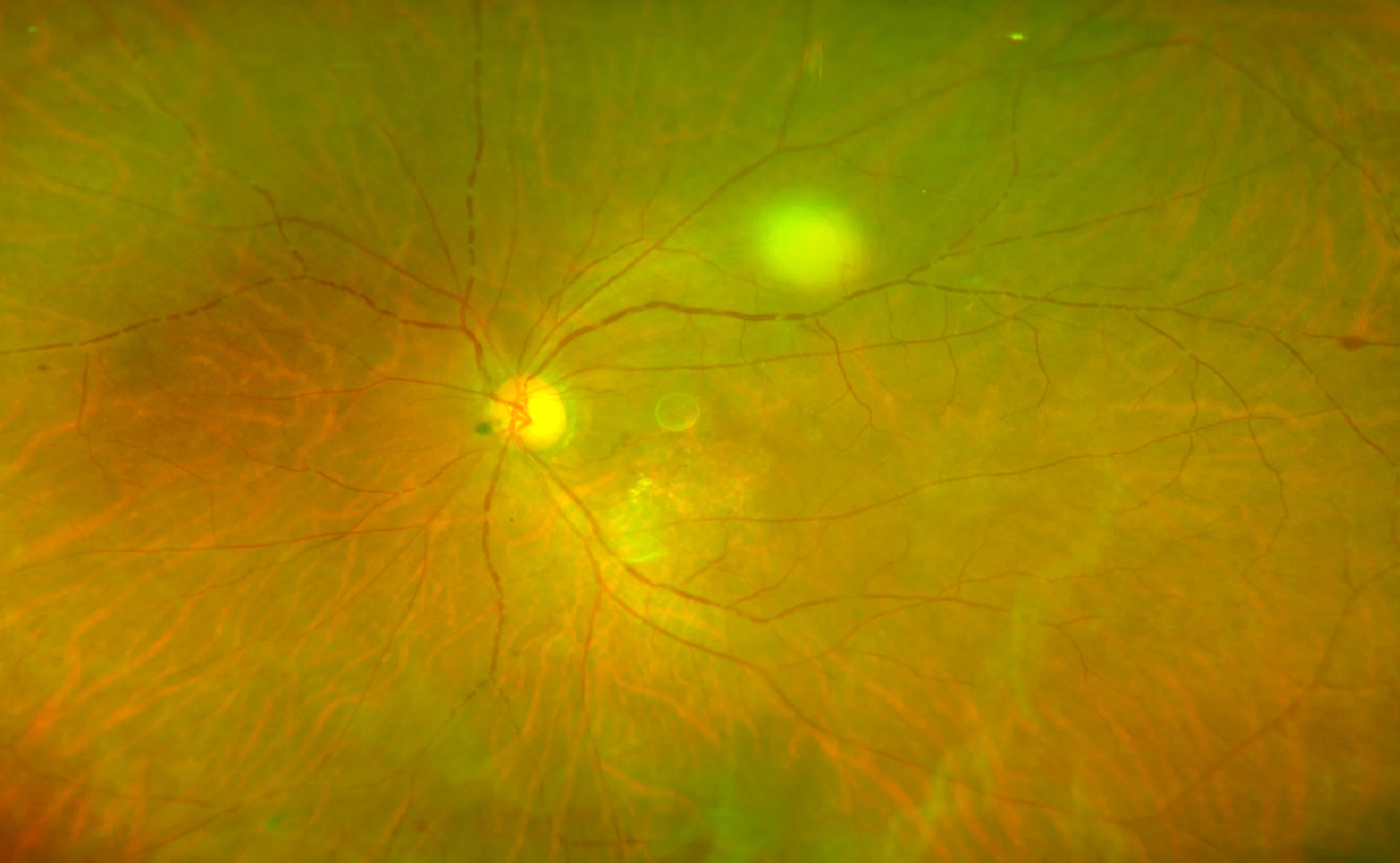

Supplement: Supplementary file 1 — Supplementary Material 1 [file 10384_2024_1107_MOESM1_ESM.tif]

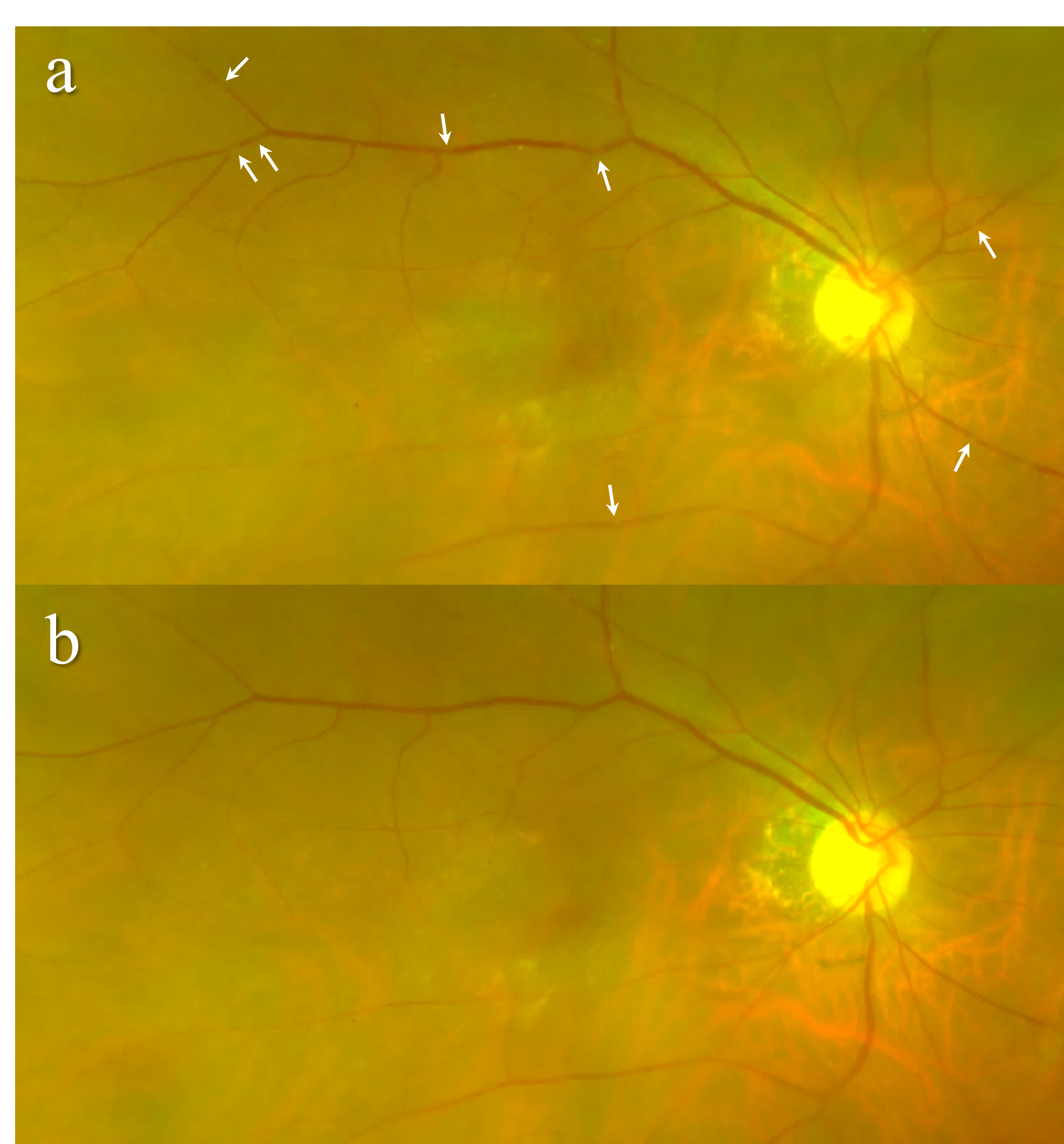

Supplement: Supplementary file 2 — Supplementary Material 2 [file 10384_2024_1107_MOESM2_ESM.tif]
