# Supplementary material for: Retinal vasculitis after intravitreal aflibercept 8 mg for neovascular age-related macular degeneration
Source: Jpn J Ophthalmol. 2024 Aug 20;68(5):531–7. doi: 10.1007/s10384-024-01107-w (PMC11420316; doi:10.1007/s10384-024-01107-w)
Supplement: Supplementary file 3 — Supplementary Material 3 [file 10384_2024_1107_MOESM3_ESM.docx]

**Supplemental Figure 1.** Widefield color fundus photograph of the left eye of an 82-year-old man with neovascular age-related macular degeneration associated with mixed type 1 and type 2 macular neovascularization treated with an initial injection of aflibercept 8 mg. Four weeks after the treatment, intraocular inflammation associated with retinal vasculitis was observed. Best-corrected visual acuity is 0.6 (0.22 logarithm of the minimum angle of resolution units). The widefield color fundus photograph shows multiple sites of localized narrowing of the retinal vessels, especially retinal veins, and mild intraretinal hemorrhage.

**Supplemental Figure 2.** Color fundus photographs of the right eye of a 78-year-old man with previously treated neovascular age-related macular degeneration associated with polypoidal choroidal vasculopathy. At baseline, best-corrected visual acuity (BCVA) was 0.5 (0.30 logarithm of the minimum angle of resolution units). (a) Four weeks after initial injection of aflibercept 8 mg, multiple sites of localized narrowing of the retinal vessels, especially retinal veins, are observed (arrows). Aflibercept 8 mg therapy was discontinued due to the development of intraocular inflammation associated with retinal vasculitis. (b) Two weeks after a posterior subtenon injection of triamcinolone acetonide (30mg/0.75mL), the multiple sites of localized narrowing of the retinal vessels have disappeared. BCVA remained unchanged during the course.
